# Online Characterization of Internal Stress in Aluminum Alloys During Laser-Directed Energy Deposition

**DOI:** 10.3390/s25082584

**Published:** 2025-04-19

**Authors:** Yi Lu, Jian Dong, Wenbo Li, Chen Wang, Rongqi Shen, Di Jiang, Yang Yi, Bin Wu, Guifang Sun, Yongkang Zhang

**Affiliations:** 1 College of Mechanical and Electronic Engineering, Nanjing Forestry University, Nanjing 210037, China; yi_lu@njfu.edu.cn (Y.L.); d_j@njfu.edu.cn (J.D.); wenbo@njfu.edu.cn (W.L.); wangchen@njfu.edu.cn (C.W.); srqlanzhou@126.com (R.S.); jiangdi@njfu.edu.cn (D.J.); yiyang_njfu@163.com (Y.Y.); 2 School of Mechanical Engineering, Southeast University, Nanjing 211189, China; gfsun@seu.edu.cn; 3 School of Electrical and Mechanical Engineering, Guangdong University of Technology, Waihuanxi Road 100, Guangzhou 510006, China; zykseu@163.com

**Keywords:** laser-directed energy deposition, numerical simulation, residual stress, online monitoring, machine vision

## Abstract

In laser-directed energy deposition (LDED) additive manufacturing, stress-induced deformation and cracking often occur unexpectedly, and, once initiated, they are difficult to remedy. To address this issue, we previously proposed the Dynamic Counter Method (DCM), which monitors internal stress based on deposition layer shrinkage, enabling real-time stress monitoring without damaging the component. To validate this method, we used AlSi10Mg material, which has a low melting point and high reflectivity, and developed a high-precision segmentation network based on DeeplabV3+ to test its ability to measure shrinkage in high-exposure images. Using a real-time reconstruction model, stress calculations were performed with DCM and thermal–mechanical coupling simulations, and the results were validated through XRD residual stress testing to confirm DCM’s accuracy in calculating internal stress in aluminum alloys. The results show that the DeeplabV3+ segmentation network accurately extracted deposition-layer contours and shrinkage information. Furthermore, DCM and thermal–mechanical coupling simulations showed good consistency in residual stress distribution, with all results falling within the experimental error range. In terms of stress evolution trends, DCM was also effective in predicting stress variations. Based on these findings, two loading strategies were proposed, and, for the first time, DCM’s application in online stress monitoring of large LDED components was validated, offering potential solutions for stress monitoring in large-scale assemblies.

## 1. Introduction

Laser-directed energy deposition (LDED) represents an advanced technology in additive manufacturing (AM) that has attracted substantial interest in recent years [1]. This technique employs a high-power laser as the heat source, introducing powdered materials into a molten pool via a coaxial nozzle to achieve the layer-by-layer deposition of components [2]. LDED is characterized by its rapid forming capability, flexible design options, and precise control of material composition, making it well suited for producing refractory, hard-to-machine, and reactive materials with complex geometries in a near-net shape manner [3,4]. These features grant LDED extensive application potential in sectors such as aerospace [5] and biomedical engineering [6].

Nonetheless, the fast heating and cooling cycles inherent to LDED induce significant thermal stresses, often leading to sharp temperature gradients within the manufactured parts. These gradients contribute to the accumulation of internal stresses, resulting in defects such as cracks and distortions. The issue is especially pronounced in the fabrication of large-scale components [7,8]. Since stress-related defects in additive manufacturing emerge progressively without obvious precursors, mitigating such issues once they arise is particularly challenging [9]. Obtaining insights into the stress evolution of components during manufacturing can play a pivotal role in preventing stress-induced defects, reducing material waste, and offering critical guidance for controlling both the geometry and properties of components throughout the process.

Currently, the detection of residual stresses mainly relies on offline analysis, which primarily includes offline experimental measurements and numerical simulation. In terms of experimental measurements, they are mainly divided into destructive and non-destructive techniques. Destructive approaches like the hole-drilling method [10] and the contour method [11] provide insights into internal stress distributions but cause irreversible damage, contradicting the principles of additive manufacturing. Non-contact measurement methods, such as X-ray diffraction (XRD) [12,13] and neutron diffraction [14,15] can non-destructively measure internal stresses [16]. However, the equipment for these techniques is large and the detection speed is slow [17], making it difficult to match the pace of additive manufacturing. These techniques are mainly used for the detection of internal stresses in components after manufacturing. Additionally, contact-based methods like strain gauges are often rendered impractical during the process due to high temperatures, leading to frequent sensor failures [18]. Therefore, integrating traditional residual stress testing methods into the additive manufacturing process is very challenging.

In the field of numerical simulation research, most studies focus on idealized models. To improve computational accuracy, many studies have dedicated efforts to developing finite element models to optimize prediction results. Daniel et al. [19] proposed a model based on thermal–metallurgical–mechanical (TMM) coupling, which comprehensively considers the effects of thermal stress and phase transformation stress on the multi-layer selective laser melting (SLM) process, thereby improving the prediction accuracy of residual stress. J. Ahn et al. [20] developed a numerical model considering non-isothermal diffusion and diffusionless solid-state phase transformation, successfully achieving precise simulation of residual stress and deformation during fiber laser welding and post-weld heat treatment of Ti-6Al-4V sheets. However, despite considerations in these models for heat source optimization, material phase transformation latent heat, and heat input changes caused by molten pool evaporation, their deposition models remain idealized and struggle to accurately reflect actual deposition morphology. Building upon this foundation, some studies have integrated fluid dynamics simulation with internal stress analysis techniques. Usually, fluid simulation is used to model the shape of the molten pool and deposition layer. Subsequently, the shape of the deposition layer formed by the natural flow of the molten pool was used as the geometric model for thermal–mechanical coupling numerical calculations, aiming to fit the real stress distribution in the component. Ma et al. [21] investigated an efficient smoothed particle hydrodynamics method for simulating laser powder bed fusion, which comprehensively considers complex boundary heat transfer and phase transformation processes in multi-physics fields. Through modified heat source models and the precise resolution of surface continuous forces, this method vividly reproduces the powder bed fusion process. Liang et al. [22] combined fluid dynamics models with finite element models, highly restoring the molten pool flow and powder spreading processes, and calculated the residual stress distribution of additive manufacturing components based on the solved temperature field results. Compared to traditional thermal dynamic finite element calculation methods, this approach significantly improves the accuracy of residual stress calculations. Although the deposition layer morphology simulated by this method approximates actual deposition effects, it still cannot fully 1:1 replicate the real processing procedure. Additionally, fluid dynamics simulations consume substantial computational resources, are time-consuming, and have limited model scalability, making it challenging to assess the dynamic changes in internal stresses during the manufacturing process.

To improve computational speed, researchers have focused on simplifying the calculation process, with the intrinsic strain method emerging as a widely recognized approach for rapid stress calculation [23]. However, most existing studies do not base their calculations on actual deposition morphology and continue to rely on offline predictions of residual stress [24,25]. Despite the continuous improvement in the accuracy of residual stress calculations through offline modeling, it is often challenging to intervene in the manufacturing process to prevent deformation or cracking of parts [26,27]. With the growing demand for intelligent manufacturing, real-time monitoring of component stress states has become a key research focus in the field of additive manufacturing. Chen et al. [24] used fiber Bragg grating sensors to monitor substrate deformation during deposition. They established a relationship between substrate strain and stress evolution in the deposition layers, thereby enabling the prediction of the trend in deposition stress changes. Zeng et al. [28] employed three-dimensional digital image correlation (3D-DIC) to capture the deformation field of the substrate in real time. They also used changes in the substrate strain to predict the stress evolution trends in the deposition layers above. The above two methods are only applicable to cases where the substrate is relatively thin. However, as layer deposition progresses, the influence of the substrate diminishes, significantly reducing the method’s accuracy. Wang et al. [29] employed digital image correlation for in situ monitoring of the full-field deformation of the deposition layer. However, this method requires halting the deposition process to apply artificial speckle, which is inefficient and environmentally damaging. Currently, there is still a lack of an online monitoring method that can directly reflect the stress evolution within the deposition layer.

In previous studies, we proposed a method based on the phenomenon of deposition-layer shrinkage for monitoring internal stress—the Dynamic Counter Method [30]. However, the validation of the DCM remains insufficient, with previous studies primarily focusing on single-track 316L and Inconel 718 materials. These materials exhibit lower reflectivity during deposition, and the exposure levels in the monitoring images are also low. Additionally, the applicability of DCM under different process parameters for the same material requires further investigation. To further validate the effectiveness of the DCM, this study employs an improved image segmentation network algorithm to process deposition images of AlSi10Mg material under high reflectivity and exposure conditions, accurately extracting the morphology and shrinkage information of multiple deposition layers. Based on the segmentation results, we apply DCM and thermal–mechanical coupling simulations to analyze the evolution history of internal stresses and the distribution of residual stresses in the AlSi10Mg deposition process at different scan speeds. These simulation results are then validated through XRD residual stress testing for accuracy. Additionally, to meet the computational requirements for components of varying sizes, two DCM loading strategies were proposed, which can quickly assess the internal stress state in large-scale components.

## 2. Experimental Methodology

The powder used in the experiment is AlSi10Mg (AlSi10Mg Powder, Asia New Materials Co., Ltd., Beijing, China), with a particle size range of 53–150 μm. The detailed chemical composition is provided in Table 1. The substrate material is AlSi10Mg plate, with a thickness of 10 mm. Prior to the LDED experiments, the surface was mechanically polished and cleaned with ethanol to remove impurities and oil stains.

**Figure 1:**
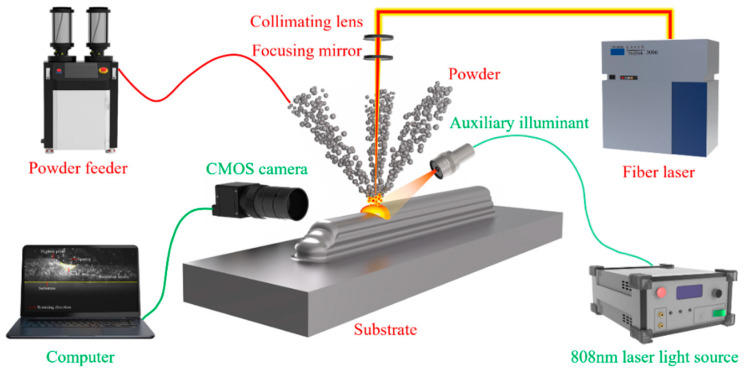
Schematic of online monitoring system of the LDED process.

Figure 1 illustrates the experimental setup for the online monitoring of the LDED process. The experiments were performed using an LDED system that featured a fiber laser with a Gaussian beam profile (TruDiode 3006, TRUMPF, Ditzingen, Germany), a coaxial powder-fed deposition head (YC52, Precitec, Gaggenau, Germany), a powder feeder (RC-PGF-D, Raycham, Nanjing, China), argon gas cylinders, and a four-axis CNC machine (AFS-1200.80, Longyuan AFS, Beijing, China). The fiber laser emitted light at a wavelength of 1030 nm, with a maximum output power of 3000 W, operating in a rectangular waveform during the experiments. The laser beam was collimated and focused through lenses with focal lengths of 100 mm and 300 mm, yielding a final spot diameter of approximately 1 mm, as measured experimentally. Metal powder was fed into the molten pool for deposition via a ring-shaped nozzle using carrier gas.

An active vision sensor monitoring system was developed to capture the specimen’s contour during deposition and track its shrinkage variations before and after deposition. The system includes a CMOS camera (Chameleon3 USB3, Teledyne FLIR, Goleta, CA, USA), a 15W continuous 808 nm laser (FC-808, CNI, Changchun, China), and a personal computer. The camera was securely mounted to the laser deposition head using a custom fixture, allowing fine adjustments along the XYZ axes and R axis to ensure the substrate remained horizontally aligned within the camera’s field of view. The 808 nm, 15 W laser provided continuous illumination to the area behind the molten pool, helping the camera clearly capture the contours of the deposition layers. To improve visibility of the molten pool, which was often obscured by bright plasma plumes, the setup included an 808 nm narrow-bandpass filter (NBF), a neutral density filter (ND), and a transparent glass protective lens (K9) in front of the camera lens. The personal computer remained connected to the camera throughout the monitoring process to facilitate continuous data acquisition.

In the experiment, a laser power of 2500 W was used with varying scanning speeds (300 mm/min, 400 mm/min, and 500 mm/min) to prepare AlSi10Mg single-track samples with five layers, each approximately 30 mm in length. The optimized process parameters include a carrier gas flow rate of 5 L/min, a powder disc speed of 0.5 r/min, and a protective gas flow rate of 15 L/min. The scanning strategy followed a unidirectional pattern. After the deposition of each layer, both the laser and powder feeding systems were switched off, and the laser head was raised by 1 mm to the preset height. The laser head would then return to the starting point of deposition, capturing clear contour data during the return. Since the system does not have height compensation, there is a slight difference between the height increment in the deposited layer and the lifting height of the laser head during the manufacturing process, which may cause defocus. Therefore, to improve the accuracy of temperature field reconstruction and to comprehensively evaluate the effect of laser defocusing on heat input, the diameters of the laser spot and powder spot were measured under different defocus conditions. At the focal point, the laser spot diameter was 1 mm, while the powder spot diameter was approximately 2.1–2.2 mm. When the laser was defocused by 5 mm, the laser spot diameter increased to approximately 2.15 mm, whereas the powder spot diameter remained nearly constant at 2.1–2.2 mm.

In addition, to better validate the subsequent numerical simulation results, we employed the widely used industrial X-ray diffraction method (X-350 AX X-ray stress tester, Aisite, Handan, China) to measure the residual stress on the surface of the specimen after cooling. In this study, five points on the surface of the specimen were selected for residual stress measurement. The residual stress test employed the inclined fixed φ method, using the Kα emission line of a Cr filament as incident radiation, with a 2θ scan range from 136° to 143°.

## 3. Visual Segmentation and 3D Reconstruction of Deposition-Layer Morphology

Deposition images of the LDED process at different scanning speeds were captured using the active visual monitoring system. These images contain rich geometric information about the molten pool and deposition layers. By developing and applying corresponding image processing algorithms to the deposition images, the surface contours of the deposition layer before and after cooling shrinkage are extracted, and height values are recorded. The resulting surface displacement differences due to cooling shrinkage provide data support for subsequent 3D reconstruction and finite element stress field calculations.

**Figure 2:**
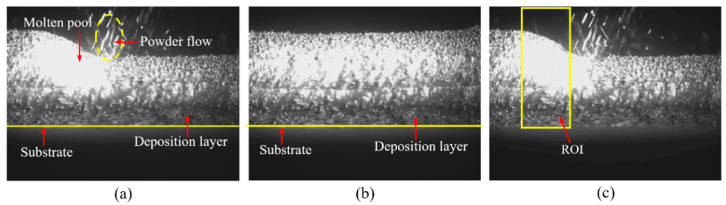
Geometry of the deposition layer. (**a**) The morphology of the deposited layers during the deposition process. (**b**) The morphology of the deposited layers after the deposition process is completed. (**c**) Schematic diagram of the region of ROI area.

Figure 2a–c show images of the deposition layer and post-shrinkage captured during the LDED process. During processing, the 808 nm external light source irradiates the surface near the molten pool, causing strong radiation from both the molten pool and deposition-layer surfaces. This results in blurred boundaries between the molten pool and the deposition layer. Additionally, the presence of powder and splatter above the molten pool introduces interference, complicating the extraction of the overall contour. For the images captured after processing, the laser and powder feeder are both turned off, providing a clear contour of the deposition-layer surface. However, the aluminum alloy deposition sample surface is also affected by the 808 nm light source, causing strong reflection that interferes with image segmentation.

To capture the maximum shrinkage of the deposited material, it is necessary to extract the maximum height formed before the material undergoes cooling shrinkage during processing. From the processing images, the overall trend of the molten pool appears sloped, with the maximum formed height located at the tail end of the pool. Therefore, the focused region is confined to the vicinity of the molten pool’s tail, where the contour above is clearer, minimizing interference. On the other hand, since the CMOS camera captures images at a resolution of 2048 × 1536, analyzing the entire image would waste computational resources and result in excessive time consumption. Therefore, a region of interest (ROI) containing the molten pool’s tail and the freshly solidified layer was defined in the image. The width of the ROI was set to 400 pixels to ensure contour continuity between consecutive frames. In the vertical direction, the upper boundary of the ROI is aligned with the top boundary of the image, while the lower boundary is set at the substrate surface to avoid the surface contour disappearing within the ROI due to deposition height instability. The image after the ROI operation is shown in Figure 2c, and only the pixels within the ROI are selected for subsequent image processing.

To accurately extract the surface contour of the deposition layer, an image segmentation algorithm is first used to segment the deposition layer. Then, an edge detection algorithm is applied to identify its external contour. Currently, traditional image segmentation algorithms typically use threshold-based principles to extract features related to the molten pool and external contours. However, these methods are often predefined and non-learnable, making them unsuitable for complex images with high light noise encountered in actual processing. Additionally, traditional image segmentation algorithms have long computation times, making them difficult to match the manufacturing speed and unsuitable for real-time tracking of the deposition layer in online monitoring. Compared to traditional algorithms, deep learning can learn and acquire prior knowledge from images, and its object recognition and localization capabilities make it more suitable for the additive manufacturing field [31].

Deeplabv3+ is one of the leading image semantic segmentation networks at present [32], known for its smooth segmentation edges and high segmentation accuracy, which is among the top in many public datasets. However, the original Deeplabv3+ network model uses Xception as the backbone for feature extraction, which leads to issues such as a large number of computational parameters and redundancy in the backbone network.

**Figure 3:**
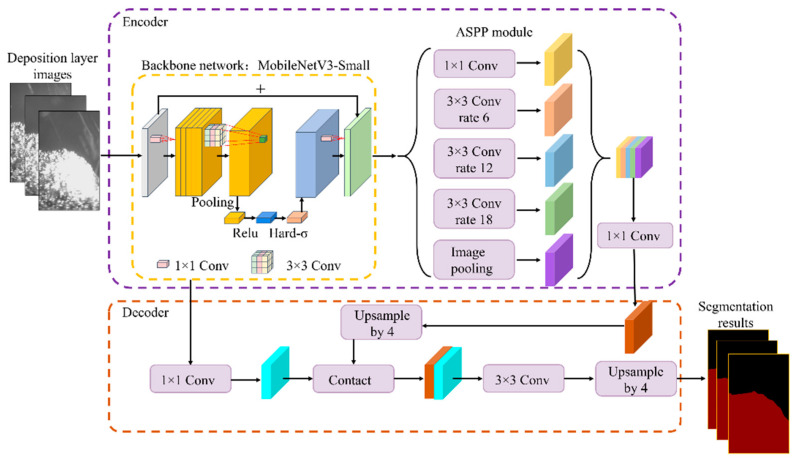
Deeplabv3+ network structure diagram.

To address this, we choose the lightweight MobilenetV3 network as the backbone for Deeplabv3+, reducing the model parameters and increasing detection speed. Additionally, MobilenetV3 introduces the Squeeze-and-Excitation (SE) module and Hard-Swish activation function, ensuring high detection accuracy. The network structure is shown in Figure 3.

The dataset required for the Deeplabv3+ model was constructed by cropping the original images, resulting in a total of 2000 images. To improve the model’s generalization ability, operations such as random rotation, random brightness adjustment, and random image noise addition were applied to the training images. After data augmentation, the original dataset was expanded to 3500 images. The final dataset was divided into a training set of 2450 images, a validation set of 700 images, and a test set of 350 images.

**Figure 4:**
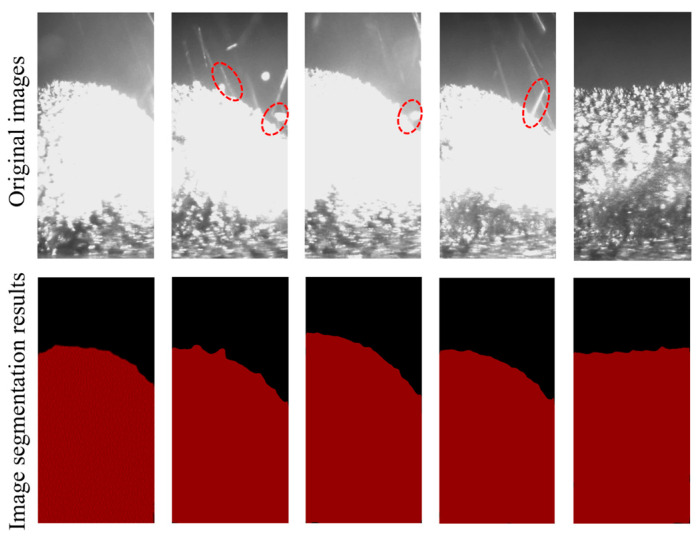
Example of image segmentation results of the Deeplabv3+ model.

Table 2 presents the training parameters used for the Deeplabv3+ model. After 100 iterations, the model’s loss function gradually stabilized and approached 0, indicating that the model had converged. During training, the model weights were saved every five iterations to facilitate the selection of the optimal model weights later. On the test set, the model exhibited high segmentation accuracy, with MIoU at 98.84%, precision at 99.41%, recall at 99.42%, and average inference time of 20 ms, demonstrating the reliability of the trained model. To visually demonstrate the model’s segmentation performance on deposition-layer images, as shown in Figure 4, three types of representative images were selected: one without interference during processing, one with splatter (shown by the red circle) during processing, and one after processing. The model’s segmentation results are shown in Figure 4. It can be seen that the model effectively removes interference such as splatter, while also achieving very precise segmentation of the surface contour.

**Figure 5:**
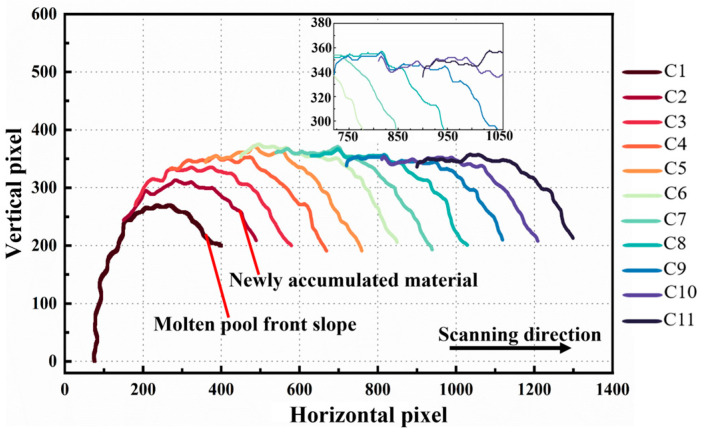
Surface profile reconstruction of the deposition layer.

Building on the Deeplabv3+ segmentation of the deposition layer, the Canny edge detection operator was applied to extract the upper surface contour of the deposition layer and obtain the corresponding pixel coordinates. Since the relative position of the camera and laser head remains fixed, and the bottom edge of the ROI corresponds to the substrate position, each vertical pixel corresponds to the actual physical height of the deposition layer in the build direction. The upper-surface contour of the deposition layer was extracted at fixed intervals, and between consecutive frames, the horizontal pixels were advanced and superimposed in an equidistant manner, gradually generating the full surface contour of the deposition layer (as shown in Figure 5). Each horizontal pixel corresponds to multiple vertical pixel values, with the maximum pixel value at the deposition location selected as the vertical coordinate for that point, indicating that the position is at the moment when the material has just finished melting and is about to begin cooling and shrinking. Similarly, all deposition-layer images for the current layer were processed to obtain their contour information. Then, by combining the unit pixel size manually calibrated in the experiment, the contour information in the image coordinate system was further converted into actual height data. Similarly, during the reverse scanning process, when the deposition layer has completed its shrinkage and the contours overlap, the same method can be applied. Finally, the contours during the processing of each layer were matched with the post-processing contours along the horizontal coordinate, and interpolation was performed along the vertical coordinate to obtain the surface displacement difference for each layer.

**Figure 6:**
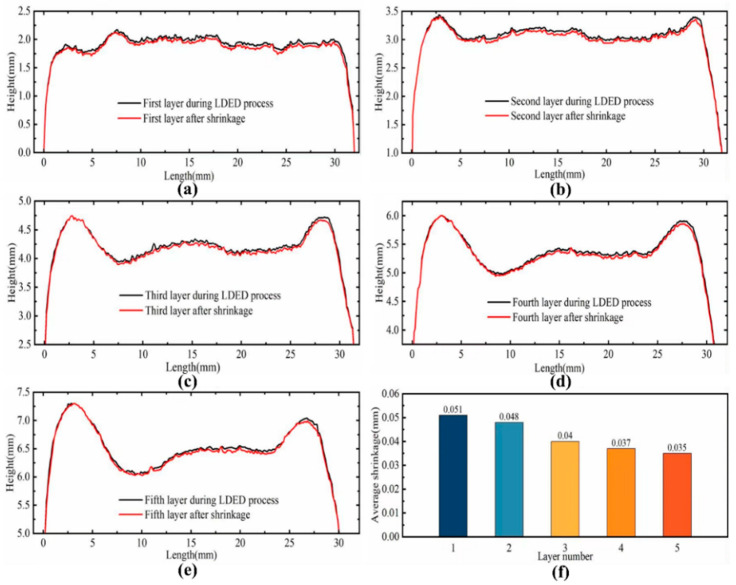
(**a**–**e**) Machine vision extracts the top profile of the 5-layer deposition layer before and after shrinkage and (**f**) the average shrinkage value of each layer.

Figure 6a–e illustrate the height variation in each deposited layer before and after cooling contraction. It can be observed that with the increase in the number of deposited layers, there is a significant over-accumulation phenomenon at both ends of the thin-walled structure. The primary cause of this phenomenon is that the start and end positions of each layer’s deposition process coincide with the machine’s startup and shutdown phases. The acceleration changes during these start–stop processes lead to excessive powder accumulation at both ends, resulting in an uneven distribution of the deposited layer, which, overall, exhibits a distribution with higher elevations at the ends and a lower elevation in the middle. On the other hand, due to the lack of height compensation, as the deposition height increases, the focal point of the powder and heat source gradually moves away from the previous deposition layer, resulting in a defocusing phenomenon. The utilization rate of the powder decreases accordingly, leading to the inability of the deposition layer to reach the preset height, and the degree of defocusing further intensifies with the increase in layers. From Figure 6f, it can be seen that the shrinkage of each layer after deposition shows a gradually decreasing trend. This phenomenon is mainly attributed to the defocusing effect, which leads to a decrease in the deposition height and deposition volume of each layer. Under the same thermal expansion conditions, the corresponding shrinkage gradually decreases due to the decrease in deposition volume.

**Figure 7:**
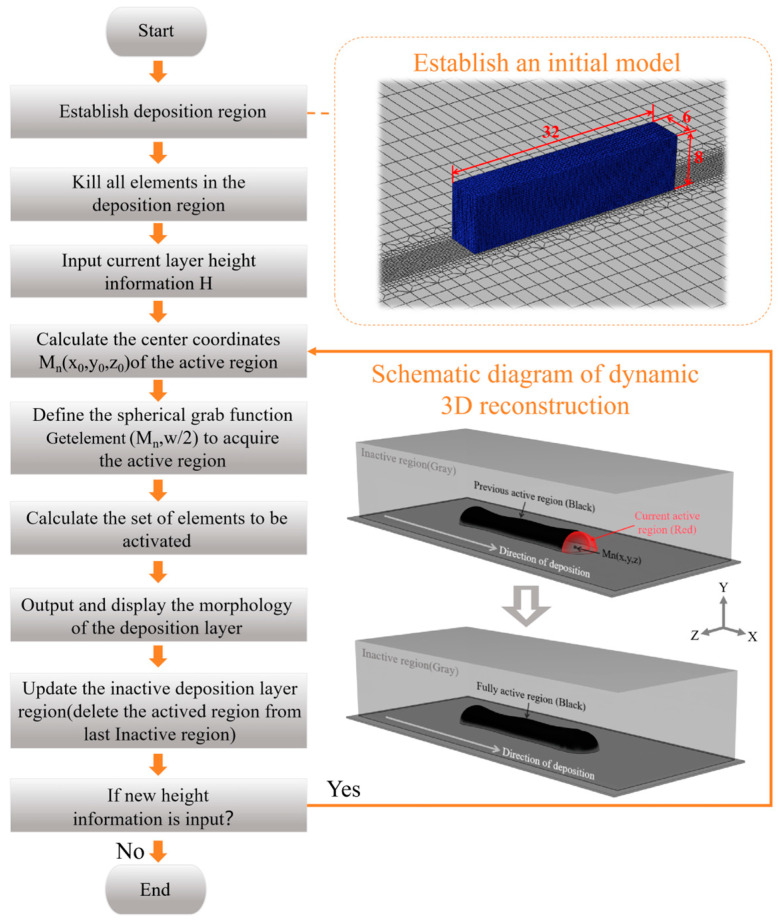
The flow chart for 3D reconstruction of deposition layer.

Based on the molten pool morphology data obtained above, real-time 3D reconstruction of the deposition-layer morphology is performed. This method combines the deposition-layer height data with molten pool position information, using Boolean modeling principles to reconstruct the geometric shape of the deposition layer [33]. To restore the natural molten pool shape formed at the front of the deposition layer due to surface tension, the deposition-layer cross-section is assumed to be arc-shaped. The X-axis represents the scanning direction, the Y-axis represents the deposition height direction, and the Z-axis represents the cross-sectional direction of the deposition layer. As shown in Figure 7, the computational domain consists of two parts: the substrate and the deposition region. The deposition region, which is used to reconstruct the morphology of the deposition layer, must be large enough to support the completion of the 3D reconstruction. Therefore, a rectangular area with dimensions of 32 mm × 6 mm × 8 mm is constructed above the substrate as the deposition zone. As shown in the Figure 7, the system receives the center coordinates Mn (x0, y0, z0) of the current position of the molten pool and the corresponding height information H. Based on the relatively stable deposition width w in the experiment, define a spherical grasping function (Mn (x, y, z), w/2), where Mn (x, y, z) represents the center of the spherical grasping function and w represents the grasping radius, and y = H − w/2. The function identifies elements in the computational domain as newly generated molten pool regions, centering them at Mn (x, y, z) with a capture radius of w/2, and then activates them. This step not only ensures a relatively stable channel width for the deposition layer but also controls the shape and height information of the molten pool to conform to the actual shape. An important additional step is that each time height information is read and the molten pool is activated, the system removes the activated elements from the nonactivated region and updates the remaining area to serve as the new nonactivated region for the next molten pool activation. Finally, the system checks for new height information to determine whether the deposition process has ended. If new height data are available, the nonactivated mesh elements are treated as the new deposition region, and the spherical grab function continues until no new deposition layers are generated.

## 4. Model Description

**Figure 8:**
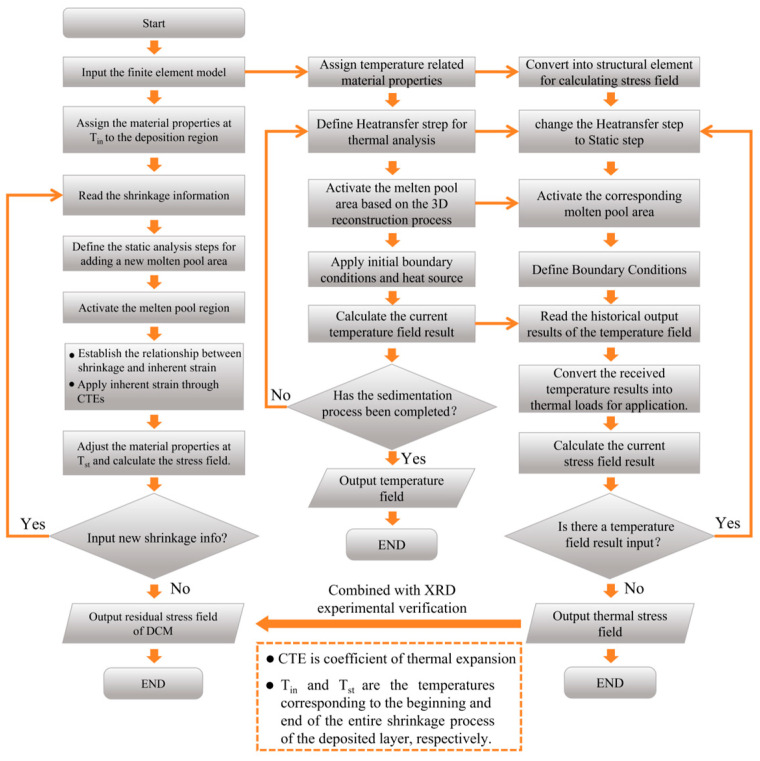
Flow chart of DCM and thermal–mechanical coupling method.

This section describes two numerical simulations based on the 3D reconstruction of the molten pool morphology: a rapid internal stress calculation using the DCM and a traditional thermal–mechanical coupling internal stress calculation based on the actual morphology (the physical model remains consistent with the previous discussion, so it will not be repeated here). Both simulations rely on a 3D reconstruction model of the deposition layer based on in situ measurements. The thermal–mechanical coupling model is calibrated using X-ray residual stress test results and is used to verify the accuracy of the DCM. The numerical computation process is shown in Figure 8. First, a 3D geometric model of the deposition process is constructed based on online monitoring data. Next, the system reads the shrinkage data caused by material cooling. By reading the position coordinates of the deposition-layer shrinkage information, the system locates the current molten pool region and activates the corresponding set of elements. It then assigns the high-temperature material properties (material properties at T_in_) to the currently activated material. Furthermore, the inherent strain (detailed in Section 4.1) is calculated based on the online-collected volume changes in the deposition layer, and the equivalent strain method is applied to the activated molten pool elements. Subsequently, the material properties after shrinkage are assigned to the currently activated material (material properties at T_st_), achieving the application of shrinkage strain. Finally, perform stress balance calculations and output the current deposition stress and deformation. Repeat this process until the system is unable to read the next set of contracted data. After completion, the system outputs the complete residual stress field of DCM.

The steps for thermal–mechanical coupling simulation based on the actual deposition morphology are shown in the right half of Figure 8. First, temperature-dependent material properties are assigned to the mesh model, and the molten pool is activated sequentially based on the 3D reconstruction process. Then, a heat source is applied to each activated molten pool region, and the temperature field distribution at each time step is calculated. This process is repeated until the deposition is complete, resulting in the temperature field distribution for the entire deposition process. Next, the stress field is calculated by reading the historical temperature field distribution for each step of the cycle. The stress field evolution during each step of the molten pool progression is computed under boundary constraints. This process is repeated until deposition is complete and the material cools to room temperature, resulting in the final residual stress field distribution. In addition, this study uses XRD to measure the surface residual stress of the component after the deposition layer has cooled. The measured stress is then used to calibrate the stress field results from the thermal–mechanical coupling numerical simulation. Finally, the internal stress calculation results of DCM are validated through a combination of XRD experiments and thermal–mechanical coupling simulations.

### 4.1. Assumptions and Theory of the Dynamic Contour Method

Previous studies have demonstrated a direct relationship between the actual shrinkage of the deposition layer and stress resolution. This study adopts the same assumptions: (1) the molten pool is stress free in its liquid state; (2) during the cooling process from the fully molten state to the solid state, the stress generated inside the deposition layer can be equated to the stress caused by the compression of the liquid pool surface to the surface profile at the completion of cooling within the deposition layer. This stress accumulation process can be expressed as [30]:

(1) $$\begin{array}{c} \sigma \left(A\right)\left(x,y,z\right)=\sigma \left(B\right)\left(x,y,z\right) \end{array}$$

Here, A represents the stress state after the molten pool undergoes cooling and shrinkage, which is the stress to be solved. B represents the stress variation stored during the process where the molten pool surface is forcibly compressed to a steady state.

In LDED, the total strain generated within the deposition layer includes elastic strain, plastic strain, thermal strain, and phase transformation strain. Therefore, the total strain can be expressed as [34]:

(2) $$\begin{array}{c} \left\{\varepsilon \right\}=\left[L\right]\left\{u\right\}=\left\{\varepsilon^{El}\right\}+\left\{\varepsilon^{Pl}\right\}+\left\{\varepsilon^{Th}\right\}+\left\{\varepsilon^{Ph}\right\} \end{array}$$

Here, $\left\{u\right\}\;$ represents the displacement; $\left[L\right]$ is the matrix formed by the differential operator connecting displacement and total strain. Among all these different kinds of strain, stress is assumed only in relation to the elastic strain [35]. Therefore, the above equation can be rewritten as:

(3) $$\begin{array}{c} \left\{\sigma \right\}=\left[D\right]\left\{\varepsilon^{El}\right\}=\left[D\right]\left(\left\{\varepsilon \right\}-\left\{\varepsilon^{Pl}\;\right\}-\left\{\varepsilon^{Th}\right\}-\left\{\varepsilon^{Ph}\right\}\right) \end{array}$$

Furthermore, by combining the variational form of the governing equations, this study derives the fundamental equations of the problem using the principle of minimum potential energy as follows:

(4) $$\begin{array}{c} \frac{\delta \Pi }{\delta u}=\frac{\delta }{\delta u}\left\{\underset{V}{∭}A\left(\varepsilon^{El}\right)dV-\underset{V}{∭}\left\{f_{v}\right\}\left\{u\right\}dV-\iint_{S_{p}}\left\{p_{s}\right\}\left\{u\right\}dS\right\}=0 \end{array}$$

In this equation, $f_{v}$ represents the body force and $p_{s}$ represents the surface force. Since no external forces are applied during the LDED process, the elastic strain energy is retained in the middle of the equation and can be expressed as:

(5) $$\begin{array}{c} \frac{\delta \Pi }{\delta u}=\frac{\delta }{\delta u}\left\{\frac{1}{2}\underset{V}{∭}{\left\{\varepsilon^{El}\right\}}^{T}\left[D\right]\left\{\varepsilon^{El}\right\}dV\right\}=0 \end{array}$$

Combining Equations (2), (3) and (5), the equation can be written as [33,35]:

(6) $$\begin{array}{c} \frac{\delta \Pi }{\delta u}=\underset{V}{∭}{\left[L\right]}^{T}\left[D\right]\left\{\left[L\right]\left\{u\right\}-\left\{\varepsilon^{Pl}\right\}-\left\{\varepsilon^{Th}\right\}-\left\{\varepsilon^{Ph}\right\}\right\}dV=0 \end{array}$$

By applying appropriate boundary conditions, the displacement of the material can be determined through the solution of the governing equation, which, in turn, allows for the calculation of the internal residual stress within the component. Typically, when non-elastic strains can be derived from these displacements, the finite element method (FEM) is used to solve the governing equation. However, under current conditions, it is challenging to establish a physical model that adequately accounts for non-elastic strains, making the solution of such nonlinear problems complex and often resulting in a solution that does not meet the expected accuracy. In this study, an alternative approach is proposed, assuming that the material shrinkage during the LDED process is primarily driven by thermal contraction and phase transformation. In this approach, the residual stress generated is balanced by both elastic and plastic strains, leading to the inherent strain, as described by the following equation [33]:

(7) $$\underset{V}{∭}{\left[L\right]}^{T}\left[D\right]\left[L\right]\left\{u\right\}dV=\underset{V}{∭}{\left[L\right]}^{T}\left[D\right]\left\{\varepsilon^{*}\right\}dV$$

Here, $\varepsilon^{*}=\left\{\varepsilon^{T}\right\}+\left\{\varepsilon^{Ph}\right\}$. Furthermore, by establishing an appropriate constitutive model, the inherent strain field is directly applied to the finite element model, effectively addressing this issue. In the current study, the application of this inherent strain field is simplified by using the shrinkage displacement distribution observed under free boundary conditions, which is then applied to the elastic model.

To apply the strain load uniformly and efficiently, this study utilizes the equivalent strain method. By defining the thermal expansion coefficient, a specific temperature is applied to the material’s shrinkage region. This induces the material’s shrinkage strain, which can be mathematically expressed as [36]:

(8) $$\begin{array}{c} \alpha_{SS}=\left|\begin{array}{ccc} \frac{\varepsilon_{11}^{*}}{\Delta T} & \frac{\varepsilon_{12}^{*}}{\Delta T} & \frac{\varepsilon_{13}^{*}}{\Delta T}\\ \frac{\varepsilon_{21}^{*}}{\Delta T} & \frac{\varepsilon_{22}^{*}}{\Delta T} & \frac{\varepsilon_{23}^{*}}{\Delta T}\\ \frac{\varepsilon_{31}^{*}}{\Delta T} & \frac{\varepsilon_{32}^{*}}{\Delta T} & \frac{\varepsilon_{33}^{*}}{\Delta T} \end{array}\right| \end{array}$$

(9) $$\begin{array}{c} \varepsilon_{\mathrm{ij}}^{*}=\varepsilon_{ji}^{*} \end{array}$$

(10) $$\begin{array}{c} \varepsilon^{*}=\alpha_{ss}\Delta T \end{array}$$

Here, $\alpha_{SS}$ is the thermal expansion coefficient, $\varepsilon^{*}$ is the inherent strain, and ΔT is the temperature change per unit.

To calculate stress, this study introduces the DCM process, as shown in the numerical simulation flow chart of the LDED process in Figure 8. The method utilizes the deposition-layer model, which is obtained through online monitoring. During the dynamic generation of this model, the system continuously tracks the height variation at the rear end of the molten pool, converting these data into shrinkage information. By identifying the coordinates of the shrinkage data, the corresponding set of elements in the shrinkage region is activated. Once activated, the inherent strain is calculated based on the volume change, and the equivalent strain method is applied to the molten pool elements. Specifically, the thermal expansion difference between key states—before and after shrinkage—is used to quantify the magnitude of shrinkage, after which stress equilibrium calculations are performed. The current stress state and deformation are then output, and this process is iterated until the deposition is completed.

### 4.2. Theory of Thermal–Mechanical Coupling Method

To validate the accuracy of the DCM calculation results, this study also conducted a thermal–mechanical coupling simulation based on experimentally measured morphology. The control equation used for thermal analysis is given by the following equation [37]:

(11) $$\begin{array}{c} \frac{\partial }{\partial x}\left(\lambda \frac{\partial T}{\partial x}\right)+\frac{\partial }{\partial y}\left(\lambda \frac{\partial T}{\partial y}\right)+\frac{\partial }{\partial z}\left(\lambda A\frac{\partial T}{\partial z}\right)+q=Cp\left(\frac{\partial T}{\partial t}\right) \end{array}$$

Here, λ is the thermal conductivity, q is the heat supplied rate, T is temperature material density, Cp is the specific heat, and t is the interaction time. To better fit the molten pool shape, this study uses a simplified double-ellipsoid heat source model for laser input. The heat source model equation is expressed as [38]:

(12) $$\begin{array}{c} Q_{1}\left(x,y,z,t\right)=\frac{6\sqrt{3}\eta f_{1}q}{a_{1}bc\pi \sqrt{\pi }}\exp\left(-\frac{3{\left(x-x_{0}\right)}^{2}}{{a_{1}}^{2}}-\frac{3y^{2}}{b^{2}}-\frac{3z^{2}}{c^{2}}\right),x\geq x_{0} \end{array}$$

(13) $$\begin{array}{c} Q_{2}\left(x,y,z,t\right)=\frac{6\sqrt{3}\eta f_{2}q}{a_{2}bc\pi \sqrt{\pi }}\exp\left(-\frac{3{\left(x-x_{0}\right)}^{2}}{{a_{2}}^{2}}-\frac{3y^{2}}{b^{2}}-\frac{3z^{2}}{c^{2}}\right),x\leq x_{0} \end{array}$$

Here, $Q_{1}$ and $Q_{2}$ are the thermal energy density, q is the heat input power, η is the powder absorption rate, and $f_{1},f_{2}$ represents the energy distribution coefficients of the two ellipsoids in the double-ellipsoid heat source model, with the relationship $f_{1}+f_{2}=2$. $a_{1}$, $a_{2}$, b and c are the shape parameters of the double-ellipsoid heat source. During the actual deposition process, the material surface exhibits random upward and downward fluctuations. To better simulate this and ensure that the heat source continuously fits the surface of the deposition layer, this study adopts a point-to-point path definition method to determine the scanning path. The heat source parameters are used to adjust the molten pool size, and the motion equation of the path is parameterized over time as:

(14) $$\begin{array}{c} r\left(t\right)=\left(1-\frac{t-t_{i}}{t_{i+1}-t_{i}}\right)r_{i}+\frac{t-t_{i}}{t_{i+1}-t_{i}}r_{i+1},t=t_{i},t_{i+1} \end{array}$$

(15) $$\begin{array}{c} r_{i}=\left(x\left(t_{i}\right),y\left(x\left(t_{i}\right)\right),0\right) \end{array}$$

Here, $r_{i}$ is the position vector at time $t_{i}$, and in the path, y is the position function of $x\left(t_{i}\right)$. This process occurs after the deposition-layer region is activated, capturing the dynamic surface changes in the deposition layer, denoted as the path $r\left(t\right)$, which is represented by the position vector $r_{i}$.

### 4.3. Material Properties

**Figure 9:**
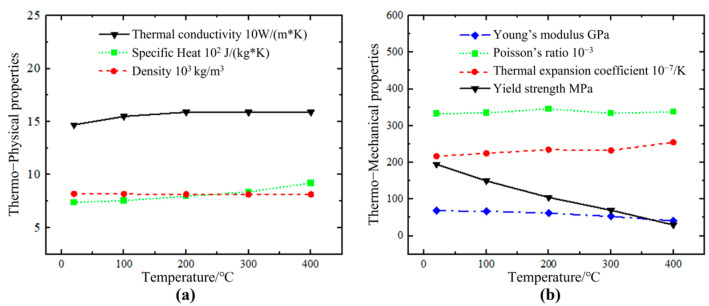
Properties of AlSi10Mg, (**a**) thermal−physical properties and (**b**) thermal−mechanical properties of AlSi10Mg.

The thermal physical and mechanical property parameters of the materials used in the simulation are shown in Figure 9. Based on the elemental composition of the aluminum alloy, the thermal physical parameters of the alloy were calculated using JMatPro7.0 software. As shown in Figure 9a, the necessary thermal physical parameters for the temperature field simulation are provided, including density, specific heat, and thermal conductivity. The aluminum alloy properties used in the experiment, shown in Figure 9, list the thermal–mechanical parameters required for stress field calculations, including the coefficient of thermal expansion, Young’s modulus, yield strength, and Poisson’s ratio.

## 5. Results and Discussion

### 5.1. Thermal–Mechanical Coupling Model Validation

**Figure 10:**
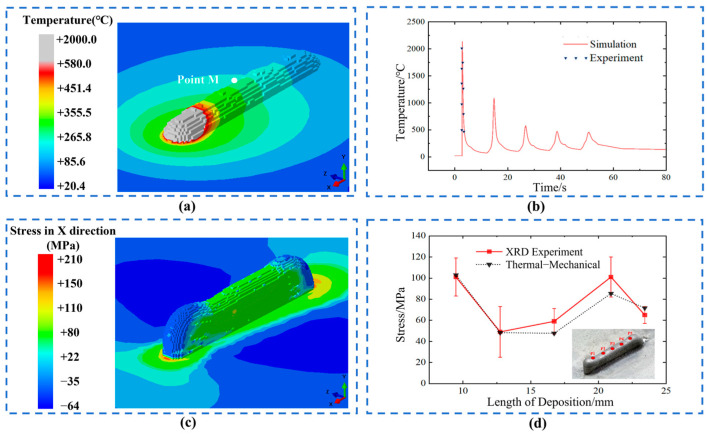
Residual stress detection and model verification for the deposition layer using parameters of 2500 W laser power and 300 mm/min scanning speed, (**a**) temperature distribution at half of the process, (**b**) comparison of the temperature history at point M with experimental data, (**c**) distribution of residual stress in the X direction, (**d**) the residual stress along the top surface of the sample.

Figure 10 presents the 3D reconstruction results and the validation of thermal–mechanical coupling simulation for a laser power of 2500 W and a scanning speed of 300 mm/min. Among them, the X-axis represents the scanning direction, the Y-axis represents the direction, and the Z-axis represents the direction parallel to the substrate plane and perpendicular to the laser heat source path, which is called the lateral direction. Figure 10a depicts the temperature field distribution during the deposition process of the first layer, as well as the position of the center point M of the deposition layer. The molten pool is elliptical in shape, with its boundary defined by the melting point of the material (580 °C). The temperature gradient and isotherm distribution in the molten pool are uneven, with a steep gradient in the front and a flat gradient in the back. Figure 10b shows the temperature history of the center point M in the first layer. As the number of deposition layers increases, the temperature at point M fluctuates periodically, while its peak temperature gradually decreases, aligning with the experimental measurement results. Figure 10c reflects the longitudinal residual stress distribution after deposition cooling is completed. The residual stress field was validated using XRD residual stress measurements at five points on the surface of the deposition layer. The locations of these measurement points are shown in Figure 10d. It shows that the distribution trend and numerical values of the thermal–mechanical coupling simulation results well match those of the XRD experimental results, demonstrating a good degree of consistency between them. In summary, the 3D reconstruction process highly restores the geometric morphology of the sedimentary layer, and based on this model, accurate temperature and stress field results are obtained, providing a dataset for subsequent DCM model validation.

### 5.2. DCM Validation and Stress Field Analysis

**Figure 11:**
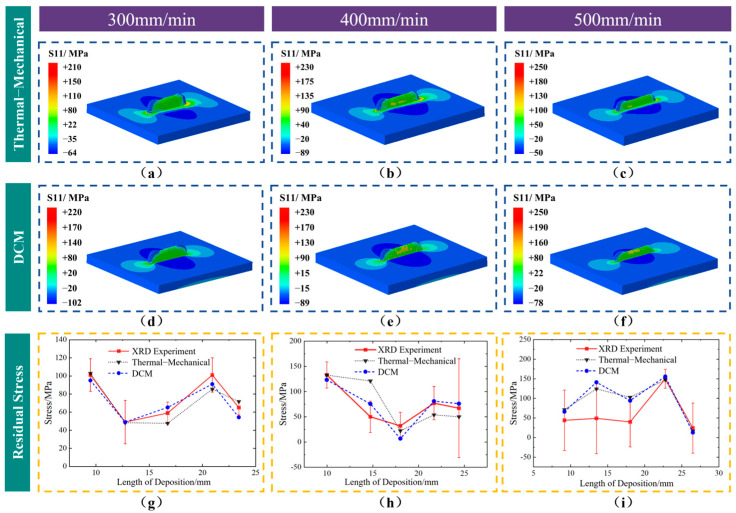
Surface stress distribution in scanning direction and experimentally measured surface stress distribution. (**a**–**f**) Comparison of surface residual stress distribution using the thermal−mechanical coupling method and DCM. (**g**–**i**) Surface residual stress.

Figure 11 compares the surface residual stress results of components obtained from DCM, thermal–mechanical coupling calculations, and X-ray diffraction (XRD) measurements at different scanning speeds. It can be observed from Figure 11a–f that the DCM results at different scanning speeds show a similar distribution to the corresponding thermal–mechanical coupling residual stress distributions. Stress concentrations appear at both ends of the interface between the substrate and deposition layer after cooling to room temperature, exhibiting tensile stress. The thermal–mechanical model results show that the upper regions at the left and right ends of the deposition layer exhibit lower compressive stress levels due to better heat dissipation and less surface mechanical constraints. This phenomenon is well reproduced in the DCM method. Furthermore, as the scanning speed increases, the maximum tensile stress in the thermal–mechanical coupling rises, showing good consistency with the residual stress results from DCM calculations. It can be observed from Figure 11g–i that the DCM surface residual stress results at different scanning speeds show trends similar to those of the thermal–mechanical coupling and experimental measurements. Most of the results fall within the experimental measurement error range, with an overall trend of decrease–increase–decrease. Overall, the DCM results effectively reproduce the stress field distribution, with the stress distribution trend consistently aligning with the actual conditions.

**Figure 12:**
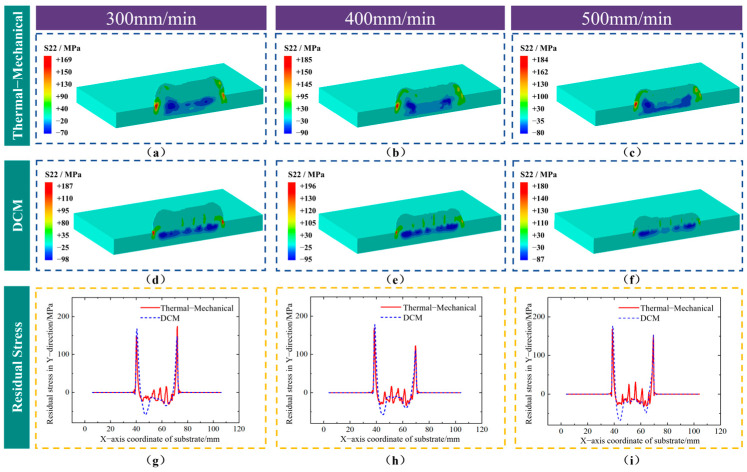
Distribution of vertical residual stress in the longitudinal section using two different methods under different scanning speeds with a laser power of 2500 W. (**a**−**c**) Thermal–mechanical coupling method. (**d**−**f**) DCM. (**g**−**i**) Stress distribution along the central axis of the interface between the substrate and the deposition layer.

Figure 12a–f show the distribution of residual stress in the vertical direction (S22) within the deposition layer. A comparison of the calculation results from both methods reveals similar distributions under different scanning speed parameters. The central region at the interface between the deposition layer and the substrate exhibits compressive stress, which increases near the ends of the deposition layer, ultimately forming areas of high tensile stress concentration at the ends of the deposition layer where it contacts the substrate. Figure 12g–i depict the stress distribution along the central axis of the interface between the substrate and the deposition layer. Owing to the numerous calculations of heat source application in the thermal–mechanical coupling methods, the internal stress distribution along this path exhibits more details compared to the DCM. However, from the perspective of the overall stress distribution, the vertical residual stress distribution along this path at different scanning speeds shows good consistency between the DCM and thermal–mechanical coupling methods. Despite some fluctuations, both methods yield similar stress levels in this region.

**Figure 13:**
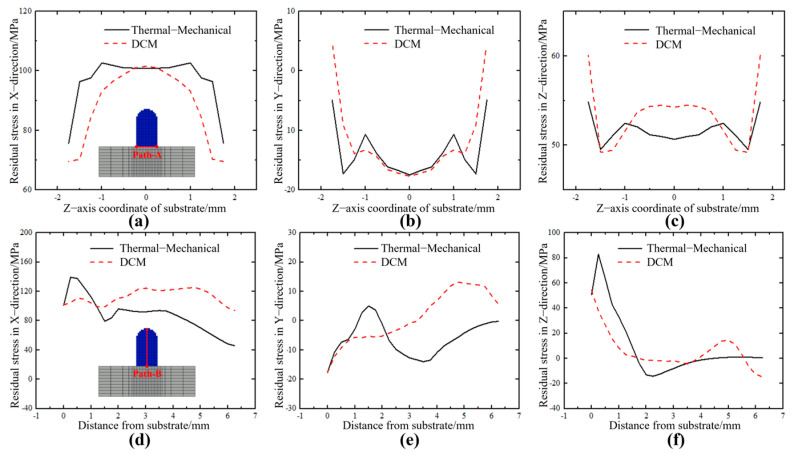
Distribution of stress in different paths in the transverse section at different scan speeds. (**a**) X component of stress along Path A. (**b**) Y component of stress along Path A. (**c**) Z component of stress along Path A. (**d**) X component of stress along Path A. (**e**) Y component of stress along Path A. (**f**) Z component of stress along Path A.

Figure 13a–c show the stress distribution trends in the x, y, and z directions along Path A at the interface between the deposition layer and the substrate in the cross-section. From the figure, it can be observed that the residual stress distributions in all directions from the DCM method show trends similar to those from the thermal–mechanical coupling calculation, with good agreement in the central region of the deposition layer. However, there is some error in the residual stress values away from the central region. This is primarily because, in the DCM model, the vertical contraction is directly obtained through visual monitoring, while contractions in other directions within the cross-section are calculated using a ninth-order linear expansion formula in conjunction with the vertical contraction strain. This led to discrepancies between the applied lateral shrinkage and the actual material volume shrinkage, resulting in the observed error. In Figure 13d–f, the residual stress distribution obtained by the DCM shows good consistency with the thermal–mechanical coupled stress distribution in all directions, especially in the stress concentration zones at the interface between the deposited layer and the substrate. However, the stress trend along Path B shown in Figure 13e exhibits slight differences in the range of approximately 1.5 mm to 3.5 mm. This discrepancy is attributed to the distinct loading methods of DCM and thermal–mechanical coupling, which results in DCM capturing fewer details compared to thermal–mechanical coupling, thereby causing certain stress fluctuations. On the other hand, in Figure 13e, the stress levels at the contact position between the deposited layer and the substrate and near the upper surface are consistent. In general, although there are some differences in the distribution along two different paths in the lateral cross−section between the DCM and thermal–mechanical coupling methods, the distribution is relatively consistent.

**Figure 14:**
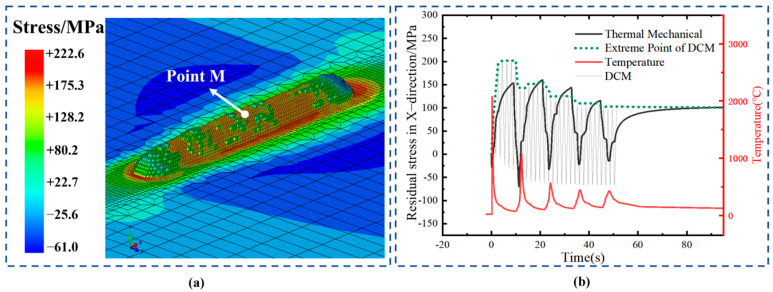
Evolution of stress in the X−direction at point M of the first layer. (**a**) Observation point M position. (**b**) Comparison of stress results between the DCM and thermal–mechanical coupling simulation.

To further verify the accuracy of DCM in stress monitoring, Figure 14 shows the stress variation curve at reference point M, which is located at the center of the first deposition layer. As can be seen from Figure 14b, the stress variation trends obtained from the two calculation methods are generally consistent. With the laser’s reciprocating scanning, the material stress at this point undergoes periodic changes. In the thermal–mechanical coupling simulation, the stress variation is negatively correlated with the temperature history at this point. When the molten pool first reaches the position of point M, the powder entering the pool melts at high temperatures, and the liquid metal inside is in a stress-free state. As the molten pool gradually moves away, the temperature at point M drops rapidly, and continuous shrinkage causes the stress near point M to rise quickly. When the next deposition layer begins and the laser approaches point M again, the remelting effect releases the internal stress near point M, causing the stress to drop quickly. When point M enters the molten pool’s influence zone, the stress decreases rapidly, returning to a lower level. In some cases, under compression from surrounding material being pushed towards the molten pool, the point even experiences a period of compressive stress. As the molten pool moves further away, it enters the solidification and cooling phase, and tensile stress resumes at a higher level. This cycle repeats throughout the deposition process, where the internal stress at this point undergoes periodic thermal relaxation. The stress extremes during the solidification and cooling phase gradually decrease with each cycle, eventually stabilizing around 100 MPa after the loss of thermal input. The stress evolution history of point M calculated by DCM shows good agreement with the thermal–mechanical coupling simulation, with both methods yielding similar numerical values and trends. Both curves indicate that during the five−layer deposition process, the influence of subsequent deposition layers on the internal stress of the first layer gradually weakens, and after deposition is complete, the residual stress values predicted by both methods converge to the same level. In Figure 14b, the stress evolution history obtained from DCM exhibits multiple fluctuations, which can be attributed to two main factors. First, the DCM method directly applies shrinkage strain for calculation. This means that each time the molten pool advances and the material begins to shrink, the DCM method adds the strain load to a certain area, after which material rebound occurs, leading to periodic stress variations. Second, compared to traditional thermal–mechanical coupling simulations, the shrinkage strain loading in DCM is more discrete, resulting in a discrete stress evolution pattern. However, the envelope of stress levels after each rebound indicates that the overall stress evolution trend predicted by DCM is consistent with that of traditional thermal–mechanical coupling simulations.

From Figure 11, Figure 12, Figure 13, Figure 14, it can be observed that DCM exhibits a good degree of consistency with the thermal−mechanical coupling simulation in predicting the internal stress distribution and evolution history. Furthermore, the residual stress predicted by DCM falls within the experimental measurement error range for residual stress (Figure 11), indicating that the proposed method for calculating internal stress during LDED based on the shrinkage phenomenon of the deposition layer is feasible. The advantage of DCM lies in its ability to create computational models based on the actual morphology of the deposition layer, thereby reflecting the internal stress state of the as-manufactured components. By adjusting the mesh size, models with varying precision can be constructed, allowing a trade−off between computational time and accuracy to adapt to different monitoring environments and research needs. For instance, to achieve higher computational accuracy, the mesh size can be set to 0.1 mm or even smaller. A 0.1 mm mesh generates millions of grid points, which, although requiring longer computation times, provides more accurate predictions of the stress distribution in the deposition layer. Conversely, for rapid assessment of internal stress during the manufacturing process, the mesh size can be increased to 0.5 mm, reducing the grid count to tens of thousands and significantly shortening computation time. In tests conducted in this study, on a laptop equipped with a CPU (Intel Core i7−14650HX, Intel, Santa Clara, CA, USA) and 16 GB of memory, the computation time for a single molten pool shrinkage was approximately 10 s, enabling internal stress online monitoring with a 10 s delay. With advancements in computer hardware and algorithm optimization, this delay can be further reduced.

**Figure 15:**
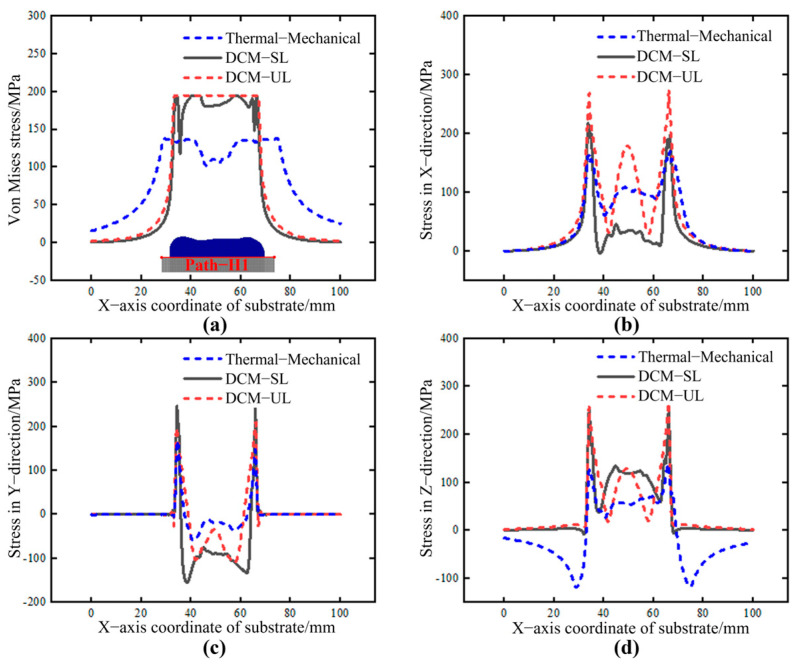
The stress distribution along Path−H1 under different loading methods. (**a**) Von Mises stress. (**b**) X component of stress. (**c**) Y component of stress. (**d**) Z component of stress.

In the field of large−scale component manufacturing, especially when using additive manufacturing technology, the massive amount of data is a challenge that cannot be ignored. Currently, it is not realistic to record all the data of each molten pool for calculation. To further enhance the computational speed of DCM and explore its adaptability to large component manufacturing, this study proposes two additional shrinkage strain loading methods: the sequential loading method (DCM−SL) and unified loading method (DCM−UL). Use the sequential loading method with each layer as a calculation cycle, obtain the shrinkage of that layer based on visual monitoring, load it into the single-layer model in one step, and calculate the stress; the unified loading method is used to obtain the shrinkage value at each moment during the entire deposited process, apply it to multiple deposition layers at once, and obtain the internal stress distribution of the area in one step. To verify the feasibility of these two rapid calculation methods, Figure 15 shows the stress results from the sequential loading method and unified loading method, with equivalent and triaxial stress curves extracted along Path H1. As shown in Figure 15a−d, the deposition layer is located between 35 mm and 65 mm in the figure. Both loading strategies show similar trends in internal stress distribution. The central region of the deposition layer−substrate contact surface generally exhibits tensile stress in the lateral and scanning directions, while compressive stress is observed in the vertical direction. Overall, while the DCM−SL and DCM−UL show some differences in the internal stress distribution at the deposition layer/substrate interface compared to the thermal−mechanical coupling method, their overall distribution patterns remain similar. This consistency underscores their significant practical value in the forming and manufacturing of large-scale complex components.

**Figure 16:**
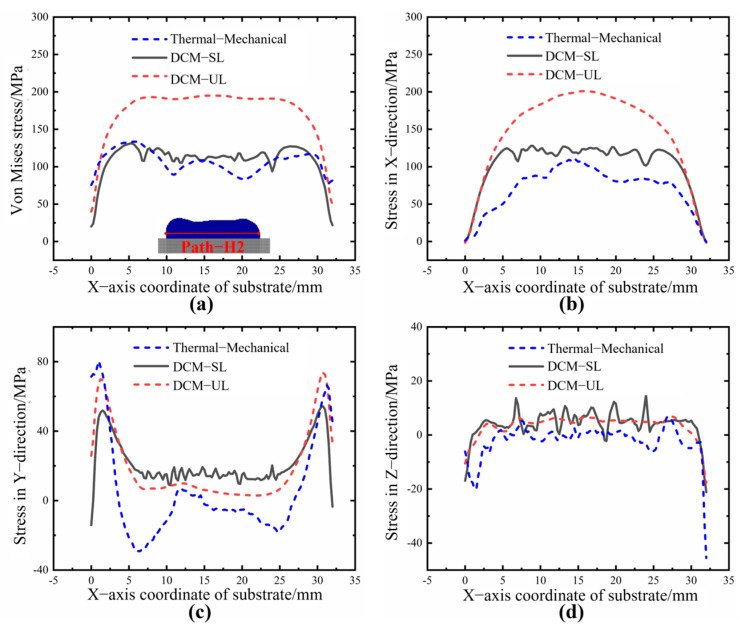
The stress distribution along Path−H2 under different loading modes. (**a**) Von Mises stress. (**b**) X component of stress. (**c**) Y component of stress. (**d**) Z component of stress.

**Figure 17:**
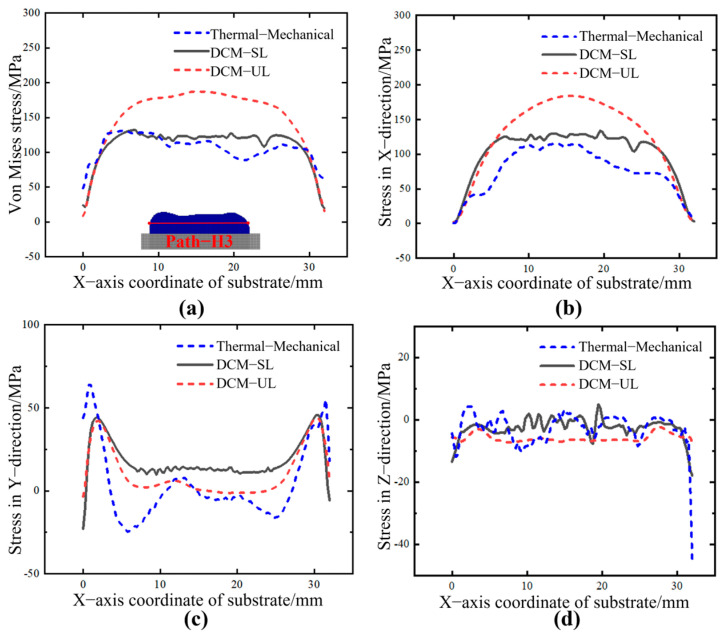
The stress distribution along Path−H3 under different loading modes. (**a**) Von Mises stress. (**b**) X component of stress. (**c**) Y component of stress. (**d**) Z component of stress.

Furthermore, the internal stress distributions along different paths in the deposition layer are extracted under two different activation methods, as shown in Figure 16 and Figure 17. Path−H2 is approximately 1.25 mm from the substrate, while Path−H3 is approximately 2.5 mm away from the substrate.

From Figure 16 and Figure 17, it can be observed that the distribution of triaxial stress and equivalent stress under two different loading methods exhibits a similar trend to the thermal−mechanical coupled stress results. Among these, the results from the DCM−SL method are closer to those of the thermal−mechanical coupling simulation, while the stress values calculated by DCM−UL are slightly higher than those from the other two methods. This difference arises because both the DCM−SL method and thermal–mechanical coupling simulations account for interlayer effects, where newly deposited material influences the stress distribution of the already-deposited material. As a result, both methods show a similar trend. In contrast, the DCM−UL method applies the shrinkage-induced strain from all deposited layers in a single step (five layers in this study), thereby weakening the interlayer interaction effects and leading to higher overall stress values. However, in terms of computational efficiency, the ranking is DCM−UL > DCM−SL > DCM. In practical manufacturing, an appropriate loading method can be chosen based on the complexity of the process. This is particularly important in the production of large-scale components; sometimes, the printing area is divided into multiple regions for separate processing. For each region, the most suitable loading method can be chosen according to specific processing requirements, ensuring a balance between local stress calculation accuracy and computational efficiency while effectively reducing hardware demands such as CPU and memory usage.

In summary, the computational efficiency of the DCM method can be optimized by adjusting mesh density and loading strategies. Particularly in the manufacturing of large components, selecting appropriate loading strategies and mesh densities enables a rapid assessment of internal stress states. By setting reasonable critical thresholds and integrating process parameter optimization strategies, deformation and cracking during manufacturing can be effectively prevented.

## 6. Conclusions

Building on previous exploration of the shrinkage phenomenon in the additive manufacturing process, this study further applies the displacement method to multi-layer deposition, introducing the “Dynamic Contour Method” (DCM) for the rapid estimation of internal stresses in LDED components. This method leverages machine vision technology to capture surface morphology changes in the deposition layer during the manufacturing process, dynamically generating reconstructed geometric models, and applying surface displacement as strain loads to the model. This allows for the quick estimation of the trend in internal stress changes and the distribution of residual stresses. The main conclusions are as follows:

(1) The constructed Deeplabv3+ semantic segmentation network successfully overcame the challenges posed by overexposure interference from powder splashing, adhesion, and the high reflectivity of AlSi10Mg material during the deposition process. It accurately extracted the deposition-layer contours and achieved a high MIoU score of 98.84%, with an average inference time of 20 ms. A multi-frame overlay method for stitching deposition-layer contours was proposed, which obtained the height and shrinkage information of the deposition layer in both molten and solidified states.
(2) DCM effectively reproduced the residual stress distribution in the single−track multi−layer samples of AlSi10Mg. The results show good consistency with the residual stresses obtained from thermal−mechanical coupling simulations and experimental measurements while keeping the prediction error within the range of experimental error. Regarding the stress evolution trend, DCM has effectively utilized the stress accumulation effect caused by the shrinkage of the melt pool, thus enabling rapid prediction of stress changes. In addition, the residual stress distribution on different cross-sections of the deposited layers shows similar patterns, which are consistent with the results of traditional thermal−mechanical coupling simulations. Moreover, the stress distribution caused by shrinkage under different scanning speeds is in good agreement with the experimental results.
(3) Two loading methods based on DCM were proposed: DCM−SL and DCM−UL. Both methods can accurately reflect the distribution of residual stress within the deposition layer. Compared with the thermal−mechanical coupling method, the DCM−SL considers interlayer stress transfer and has a more accurate distribution of internal stress, while the DCM−UL has better continuity of stress distribution within the component and high computational efficiency. Therefore, by selecting appropriate combinations of loading strategies, DCM can achieve efficient stress analysis during the additive manufacturing of large components, providing a reference for preventing stress-induced defects in components.

## Figures and Tables

**Table 1:** The chemical compositions of Inconel AlSi10Mg alloy (wt.%).

Components	Al	Si	Fe	Cu	Mn	Mg	Ni	Zn	Ti
Amount	Balance	10.03	0.12	0.01	0.005	0.36	0.005	0.008	0.35

**Table 2:** Training parameters for the Deeplabv3+ model.

Condition Parameters	Parameter Settings
The size of the image entered	512 × 512 pixel
Training batches	8
Gradient descent optimization algorithm	Adam
Momentum factor	0.9
Initial learning rate	0.0005
Learning rate update policy	Cosine annealing algorithm
Loss function	Cross entropy
The number of iterations	100

## Data Availability

The original contributions presented in this study are included in the article. Further inquiries can be directed to the corresponding authors.
